# Protocol for mitochondria lipid encapsulation using a dual-tube system for mitochondria transfer therapy

**DOI:** 10.1016/j.xpro.2025.104174

**Published:** 2025-10-30

**Authors:** Gen Hamanaka, Dong Bin Back, Ester Licastro, Shin Ishikane, Takafumi Nakano, Kazuhide Hayakawa

**Affiliations:** 1 Neuroprotection Research Laboratories, Departments of Radiology and Neurology, Massachusetts General Hospital and Harvard Medical School, Charlestown, MA 02129-2000, USA

**Keywords:** cell Biology, Metabolism, Neuroscience, tissue Engineering

## Abstract

Mitochondria transplantation therapy is emerging as a novel therapeutic approach to promote neuroprotection in central nervous system (CNS) disorders. Here, we present a protocol for mitochondrial surface modification that enhances the restoration of intracellular adenosine triphosphate (ATP) levels under conditions of oxidative stress. We describe steps for isolating mitochondria, preparing the dual-tube system, and encapsulation with lipid coat. We then detail procedures for performing assays and analyses.

For complete details on the use and execution of this protocol, please refer to Nakano et al.^1^

## Highlights

• Preparation steps for the mixture of mitochondria and lipids
• Instructions for dual-tube-mediated coating
• Handling guidance for lipid-coated mitochondria
• Procedures to enhance intracellular ATP production under oxidative stress

## Abstract (editor highlights)

Publisher’s note: Undertaking any experimental protocol requires adherence to local institutional guidelines for laboratory safety and ethics.

## Graphical abstract

**Figure:**
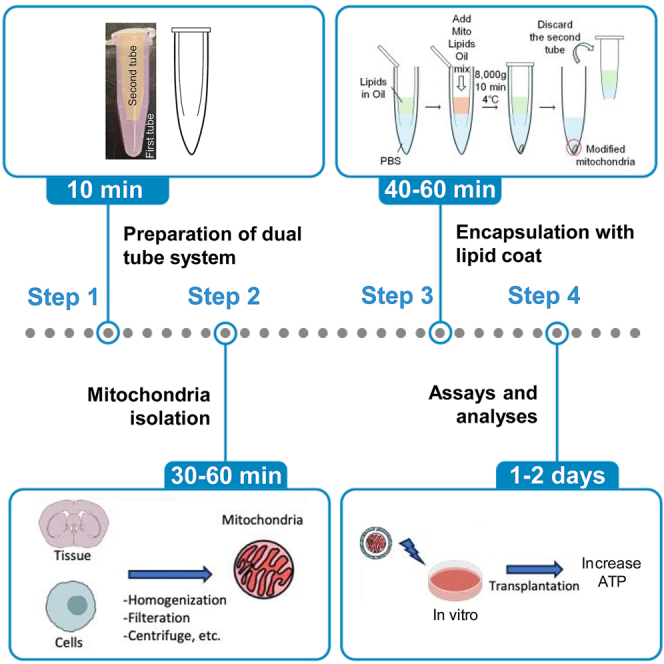

## Teaser

Mitochondria transplantation therapy is emerging as a novel therapeutic approach to promote neuroprotection in central nervous system (CNS) disorders. Here, we present a protocol for mitochondrial surface modification that enhances the restoration of intracellular ATP levels under conditions of oxidative stress. We describe steps for isolating mitochondria, preparing the dual-tube system, and encapsulation with lipid coat. We then detail procedures for performing assays and analyses.

## Before you begin

Our protocol describes a method for modifying mitochondria to enhance the therapeutic efficacy of mitochondrial transplantation. Mitochondrial transplantation is recognized as a promising strategy to restore ATP production in metabolically compromised cells. Our lab has studied the role of intercellular mitochondrial transfer and mitochondria transplantation in vivo and in vitro, where we observed mitochondrial uptake and overall efficacy.^2^^,^^3^^,^^4^^,^^5^ Although various approaches to mitochondrial modification to amplify the efficacy have been developed, our protocol offers a convenient, straightforward, and efficient method to boost ATP levels in both cellular and animal models. This strategy holds significant potential for translating basic research findings into clinical applications.

### Institutional permissions

All procedures in cell culture experiments must be approved by the Institutional Animal Care and Use Committee in accordance with regulations and guidelines. In this protocol, all experimental procedures followed the National Institutes of Health Guide for the Care and Use of Laboratory Animals and were approved by the Massachusetts General Hospital Institutional Animal Care and Use Committee (IACUC#: 2016N000153, 2016N000493).

### Material and reagent preparation for mitochondria modification

The following materials and reagents should be prepared and stored according to the manufacturer’s recommendations. Avoid repeated freeze-thaw cycles for frozen materials. Solutions and experimental tools should be sterilized by autoclaving or with 70% (v/v) ethyl alcohol (EtOH) to prevent contamination.

### Preparation of dual-tube system

**Timing: 10 min**

**Figure 1:**
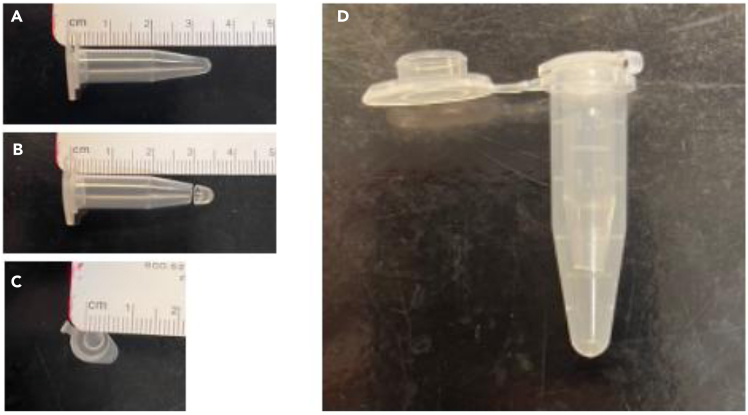
Dual tube system for MitoCoat modification The inner tube (A) is cut following our protocol (B), and its diameter is confirmed to be approximately 4 mm (C). The inner tube is then inserted into a 1.5 mL outer tube (D) to complete the setup.

The “dual-tube system” enables researchers to modify mitochondria through simple steps, making them suitable for both in vivo and in vitro applications (Figure 1). In this protocol, we use two types of microtubes (1.5 mL and 0.6 mL) from two different manufacturers; however, tubes from other suppliers can also be used, provided that the distance between them is appropriately adjusted.

1. Place one 0.6 mL tube on the bench top and cut the bottom of the tube at 3 cm from the lid with a sharp blade. The diameter of hole is around 4 mm.
2. Put the inner tube into the 1.5 mL tube.
3. Carefully add 500 μL of phosphate-buffered saline (PBS) into the inner tube, allowing it to flow through the bottom hole into the outer tube.
4. Keep the assembled dual tube system until use.

***Note:*** To avoid contamination, sanitize the blade with 70% EtOH. If you use different tubes than those shown, ensure that the surface level of PBS in the inner tube is higher than that in the outer tube.

***Note:*** Both outer and inner tubes should be autoclaved before setting.

### Mitochondria isolation

**Timing: <1 h**

Mitochondria can be isolated from various cells or tissues using established protocols suited to your specific experimental needs. For subsequent concentration measurement and surface modification, suspend the isolated mitochondria in mitochondria functioning buffer (mitobuffer) as described below. To ensure optimal therapeutic efficacy, freshly isolated mitochondria are recommended for modification. In this protocol, we also introduce the “Multiple Stepwise Separations” method, which enables the isolation of mitochondria from frozen rat placenta.

5. Place a frozen rat placenta on a 35 mm dish and add 500 μL of mitobuffer in the same dish.
6. Chop the frozen placenta into small pieces with sharp scissors.
7. Transfer the chopped placenta into grinder with mitobuffer.
  a. Rinse the dish with 500 μL of mitobuffer to collect rest of tissue on the dish.
  b. Transfer mitobuffer into grinder (total 1 mL of mitobuffer in the grinder).
8. Grind tissue slowly on ice (around 30–40 times).
9. Transfer homogenate into 15 mL tube.
  a. Rinse the grinder with 500 μL of mitobuffer to collect rest of tissue in the grinder.
  b. Transfer mitobuffer into 15 mL tube (total 1.5 mL of mitobuffer in 15 mL tube).
10. Centrifuge at 700 × *g*, 4°C, for 5 min.
  a. Collect the supernatant into a new 1.5 mL tube.
11. Centrifuge at 2,000 × *g*, 4°C, for 5 min.
  a. Collect the supernatant into a new 1.5 mL tube.
12. Centrifuge at 4,000 × *g*, 4°C, for 5 min.
  a. Collect the supernatant into a new 1.5 mL tube.
13. Centrifuge at 8,000 × *g*, 4°C, for 10 min.
14. Discard the supernatant carefully.
15. Suspend the pellet with 200 μL of mitobuffer.
16. Calculate the concentration of mitochondria using protein assay.
  a. Prepare bovine serum albumin (BSA) standards and some dilution samples of mitochondria, set reading area of 96-well plate, and record absorbance in 595 nm.

***Note:*** You can use any kinds of protein assay, such as Bradford and bicinchoninic acid (BCA) assays, and detected absorbance using 595 nm.

***Note:*** In the Step 15, if the pellet of mitochondria is small amount, adjust the volume of mitobuffer such as 50–100 μL.

## Key resources table

**Table:** 

REAGENT or RESOURCE	SOURCE	IDENTIFIER
**Antibodies**		

Total OXPHOS rodent WB antibody cocktail (1:500)	Abcam	ab110413; RRID:AB_2629281
Tomm 40 polyclonal antibody (1:1,000)	Proteintech	18409-1-AP; RRID:AB_2303725
Tom 20 (D8T4N) rabbit mAb (1:1,000)	Cell Signaling Technology	42406S; RRID_2687663
Monoclonal anti-β-actin antibody produced in mouse (1:10,000)	Sigma	A5441-.5ML; RRID:AB_476744
ECL anti-rabbit IgG horseradish peroxidase linked whole antibody (from donkey) (1:1,000)	Cytiva	NA934V
ECL anti-mouse IgG horseradish peroxidase linked whole antibody (from donkey) (1:1,000)	Cytiva	NA931V

**Chemicals, peptides, and recombinant proteins**		

1 M HEPES, pH 7.0–7.6	Sigma	H0887
Sucrose	Sigma	S0389
Sodium succinate dibasic hexahydrate	Sigma	S9637
Potassium phosphate dibasic	Sigma	P3786
ATP (optional)	Sigma	A6144
Adenosine diphosphate (ADP) (optional)	Sigma	A2754
Polyvinyl alcohol (PVA)	Sigma	360627
1,2-dodecyl-sn-glycero-3-phosphoethanolamine (DOPE)	Sigma	76548
1,2-dioleoyl-3-trimethylammonium-propane (DOTAP)	Sigma	D6182
Chloroform	Fisher	ICN19400225
Ethyl alcohol (EtOH)	Sigma	E7023
Mineral oil	Ward’s Science	470301-822
Phosphate-buffered saline (PBS), pH 7.4	Sigma	PPB006-20PAK
Tris-buffered saline (TBS)	Boston BioProducts, Inc.	BM-300
Tween 20	Sigma	P1379-500ML
Non-fat dry milk	LabScientific bioKEMIX, Inc.	M0841
NuPAGE LDL sample buffer (4×)	Invitrogen	NP0007
Sample reducing agent (10×)	Invitrogen	NP0009
Phosphatase inhibitor cocktail	Sigma	P5726-1ML
Protease inhibitor cocktail	Cytoskeleton	PIC02
Bio-Rad protein assay dye reagent concentrate	Bio-Rad	500-0006
NuPAGE MEM SDS running buffer (20×)	Invitrogen	NP0002

**Critical commercial assays**		

CellTiter-Glo luminescent cell viability assay	G7571	Promega

**Other**		

WHEATON Tenbroeck tissue grinder, 2 mL	DWK Life Sciences	357422
Microcentrifuge tubes 1.5 mL, small cap, boiling proof	Crystalgen	L-2054
0.5 mL PCR tubes with flat caps, high profile, clear	Bio-Rad	TBI0502
96-well assay plate (black plate, clear bottom with lid)	Corning	3603
iBlot 2 PVDF regular stacks	Invitrogen	IB24001
Heatblock I	VWR	13259-050
Mini gel tank	Thermo Fisher Scientific	A25977
PowerEase 300 W	Thermo Fisher Scientific	PS0300
iBlot 2 gel transfer device	Invitrogen	IB21001
G:Box	Syngene	chemi XRQ
SpectraMax	Molecular Devices	M5

## Materials and equipment

### Reagents for mitochondria modification

**Table:** 5× mitobuffer

Reagent	Final concentration	Amount
1 M HEPES, pH 7.0–7.6	50 mM	2.5 mL
Sucrose	1.25 M	21.4 g
Sodium succinate dibasic hexahydrate	25 mM	337.5 mg
Potassium phosphate dibasic	10 mM	87.0 mg
ATP (optional)	5 mM	138 mg
ADP (optional)	0.5 mM	10.5 mg
DW	–	Up to 50 mL

Store at 4°C and protect from light for up to 1 month. Dilute with DW for 1× mitobuffer before use.

**Table:** 20% (w/v) polyvinyl alcohol (PVA) stock solution

Reagent	Final concentration	Amount
PVA	10 mM	20 mg
DW	–	Up to 100 μL

Dissolve completely at a proper temperature (around 37°C).

**Table:** 100 mM 1,2-Dodecyl-sn-glycero-3-phosphoethanolamine (DOPE) solution

Reagent	Final concentration	Amount
DOPE	100 mM	100 mg
Chloroform	–	200 μL
Ethyl alcohol	–	1140 μL

Make aliquots and store at −20°C.

**Table:** 300 mM 1,2-dioleoyl-3-trimethylammonium-propane (DOTAP) solution

Reagent	Final concentration	Amount
DOTAP chloride	300 mM	50 mg
Chloroform	–	200 μL
Ethyl alcohol	–	140 μL

Make aliquots and store at −20°C.

**Table:** Suspension buffer

Reagent	Final concentration	Amount
20% PVA stock solution	1%	1 μL
DW	–	19 μL

Prepare solution buffer before use.

**Table:** Lipid mixture

Reagent	Final concentration	Amount
Mineral oil	N/A	300 μL
100 mM DOPE	1 mM	3 μL
300 mM DOTAP	1 mM	1 μL

Prepare solution before use.

**Table:** Sample buffer

Reagent	Final concentration	Amount
NuPage LDL sample buffer (4×)	N/A	350 μL
Sample Reducing Agent (10×)	N/A	100 μL
Phosphatase inhibitor cocktail	10 μM	15 μL
Protease inhibitor cocktail	10 μM	15 μL
DW	–	1 mL

Prepare solution before use.

## Step-by-step method details

This section provides a detailed protocol for mitochondria modification using artificial lipids composed of DOPE and DOTAP. This method enables researchers to conveniently encapsulate mitochondria with artificial or biological lipid components as needed —referred to as MitoCoat—for use in both cell-based and animal studies. The MitoCoat strategy offers a straightforward approach for mitochondrial surface modification, facilitating broader application in experimental and therapeutic research.

### Preparation of isolated mitochondria mixture

**Timing: 10 min**

This step is critical for efficiency of encapsulating mitochondria.

1. Measure the concentration of mitochondria, and suspend 50–100 μg (based on protein concentration) with mitobuffer into 1.5 mL tube.
  a. Centrifuge the tube at 8,000 g for 10 min.
  b. Discard supernatant completely.
2. Resuspend the pellet of mitochondria with 10 μL of suspension buffer.

***Note:*** The ratio of mitochondria/ suspension buffer is critical for this protocol. The typical ratio is 10 μg of mitochondria per 1 μL of buffer, and the total volume of the suspension buffer should not exceed 10 μL. You should calculate the concentration of mitochondria carefully in Mitochondria isolation, 16.

### Preparation of lipid mixture composed of DOPE and DOTAP in mineral oil

**Timing: 5 min**

This step describes how to prepare lipid mixture composed of DOPE and DOTAP in mineral oil (defined as lipid nanoparticle solution). Below is an example for one dual tube system.

3. Pour 300 μL of mineral oil into 1.5 mL tube, and add 3 μL of 100 mM DOPE and 1 μL of 300 mM DOTAP.
  a. Mix well using a vortex.
  b. Centrifuge at 1,000 *g* for 1 min to remove air bubbles in the oil.

### Preparation of water/oil (W/O) emulsion with mitochondria

**Timing: 5 min**

This step describes how to prepare mitochondria-lipid mixture (W/O emulsion).

4. Pour 200 μL of lipid nanoparticle solution (step 3) into a new 1.5 mL tube.
5. Add 10 μL of mitochondria in suspension buffer to 200 μL of lipid mixture.
  a. Pipette gently around 10 times.

### Encapsulation of mitochondria in lipid nanoparticles

**Timing: 20 min**

In this step, mitochondria are encapsulated with artificial lipids in preparation for transfer experiments. PBS is used instead of mitobuffer to eliminate the influence of externally added ATP or ADP, which could otherwise stimulate endogenous mitochondria within the recipient tissue or cellular environment. This precaution is critical to ensure that any observed mitochondrial function is attributable solely to the transplanted mitochondria, as neither ATP nor ADP is present during the encapsulation process.

6. Pour 100 μL of the lipid nanoparticle solution (step 3) onto PBS in the inner tube carefully.
  a. Confirm the lipid nanoparticle layer is properly layered on the PBS.
7. Add 200 μL of W/O emulsion (step 5) onto lipid nanoparticle layer in the inner tube carefully.
  a. Close the lid of the inner tube.
8. Centrifuge the dual tube at 8,000 *g* for 10 min.
  a. Remove the inner tube containing the oil layer carefully.
  b. Discard the supernatant in the outer tube.
9. Suspend the pellet with PBS to the desired volume.

***Note:*** The suspension volume should be adjusted according to the experimental design for mitochondrial treatment in vitro. In our setup, we typically apply 5–10 µg of encapsulated mitochondria per well in a standard 96-well plate.

***Note:*** We recommend using nanoparticle-encapsulated mitochondria immediately after preparation. Nevertheless, limited data suggest they may remain stable for up to two days at −80°C. (see also in “limitations”).

### (Optional 1) western blot analysis to confirm the state of MitoCoat (lipid-coated mitochondria)

**Timing: <2 days**

To confirm whether lipid coating does not damage mitochondrial function, we assess the protein expressions (such as OXPHOS, Tomm 40, Tomm 20 etc.) using Western blotting.

The following protocol is an example of how to do the Western blotting on MitoCoat.

10. Dissolve MitoCoat (from step 9) with appropriate volume of sample buffer.
  a. Dissolve non-coated mitochondria with sample buffer as a control.
  b. Heat at 85°C for 10 min.
11. Apply 15–20 μL of samples in each well, and run electrophoresis (PowerEase 300 W) at 100 V for 60 min.
  a. In the meantime, prepare 5% skim milk in TBST for a blocking buffer.
12. Transfer proteins from gel to membrane using iBlot 2 for 7 min.
  a. Place gel on the PVDF membrane carefully to avoid including air bubbles between membrane and gel.
  b. Select P0 (7 min) in the “Preset Template”.
13. Block the membrane with 5% skim milk in TBST at ambient temperature for 1 h on the shaker.
  a. Meanwhile, dilute primary antibodies using 1% skim milk in TBST.
14. Add diluted primary antibody.
  a. Incubate at 4°C for around 15 h.
15. Wash the membrane with TBST at ambient temperature for 10 min, 5 times.
  a. Meanwhile, prepare secondary antibody diluted with 1% skim milk in TBST.
16. Add diluted secondary antibody.
  a. Incubate at ambient temperature for 1 h.
17. Wash the membrane with TBST at ambient temperature for 10 min, 5 times.
  a. During the fifth wash, prepare SuperSignal West Pico PLUS Luminol/Enhancer.
18. Treat the membrane with SuperSignal West Pico PLUS Luminol/Enhancer on the plastic wrap for 1 min in dark.
19. Place the membrane in the G:Box, detect signals (exposure time; 30 s, 1 min, 3 min, 5 min, 8 min, 10 min, 15 min, and 20 min), and save images as jpeg for further analysis.

**Table:** 

Primary antibodies	Dilution
Total OXPHOS Rodent WB Antibody Cocktail	1:500
Tomm 40 Polyclonal antibody	1:1,000
Tom 20 (D8T4N) Rabbit mAb	1:1,000
Monoclonal Anti-β-Actin antibody produced in mouse	1: 10,000

Secondary antibodies	Dilution
ECL Anti-Rabbit IgG Horseradish Peroxidase linked whole antibody (from donkey)	1:1,000
ECL Anti-mouse IgG Horseradish Peroxidase linked whole antibody (from donkey)	1:1,000

***Note:*** If you are supposed to examine the expression of OXPHOS, do NOT heat the samples in step 10, b.

***Note:*** When transferring protein from gel to membrane, choose an appropriate membrane—PVDF or nitrocellulose—depending on the antibody and detection method. In particular, when using the anti-OxPhos antibody, we recommend using a PVDF membrane for optimal signal quality.

***Note:*** Here, we use SuperSignal™ West Pico PLUS Luminol/Enhancer for chemiluminescent detection. This reagent can sometimes produce stronger-than-expected signals, so it is advisable to adjust the enhancer or exposure time as needed to achieve optimal detection of the target protein.

***Note:*** You can use any imaging system capable of detecting luminescence. If such an imager is not available, X-ray film can be used as an alternative to visualize the signal.

### (Optional 2) application of MitoCoat (lipid-coated mitochondria) in treatment experiments

**Timing: <2 days**

**Figure 2:**
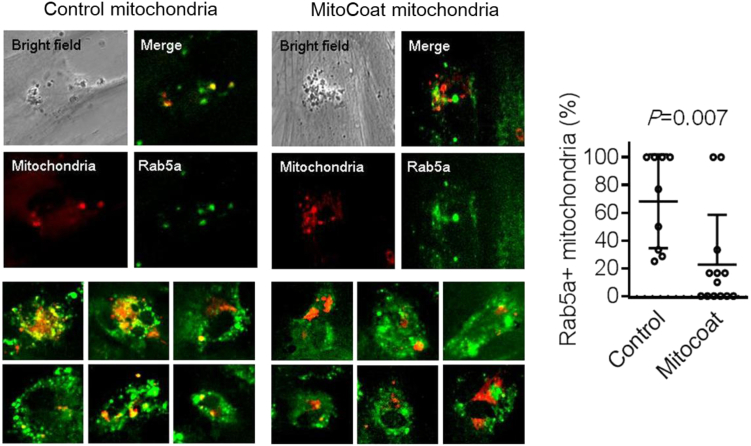
Assessment of mitochondria incorporation into cells Mitochondria were isolated from human astrocytes and used either in their native form or after coating with DOTAP and DOPE dissolved in mineral oil, referred to as MitoCoat mitochondria. Both control (uncoated) and MitoCoat mitochondria were labeled with MitoTracker Deep Red (100 nM, M22426, Thermo Fisher Scientific) for 20 min, followed by washing to remove unbound dye from the mitochondrial suspension. The labeled mitochondria were then added to Rab5a-GFP–positive human brain endothelial cells (C10586, Thermo Fisher Scientific) and incubated for 1 h to visualize mitochondrial incorporation. While incorporation was observed in both groups, MitoCoat mitochondria showed significantly reduced colocalization with early endosomes (n = 13) compared to control mitochondria (n = 9), suggesting that MitoCoat mitochondria may be incorporated via a distinct pathway, such as membrane fusion mediated by DOPE. Quantification of colocalization between Rab5a and Deep Red–labeled mitochondria revealed a statistically significant difference (p = 0.007, unpaired two-sided t-test). Data are presented as mean ± SD.

**Figure 3:**
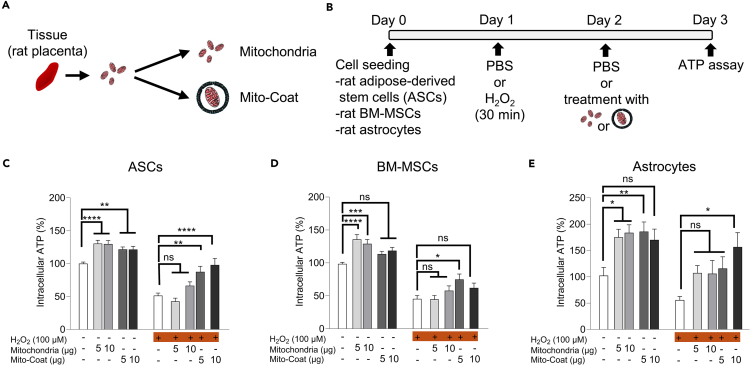
Lipid-coated mitochondria effectively restore ATP levels in cells subjected to H_2_O_2_-induced oxidative stress (A) Mitochondria were isolated from rat placenta and used either in their native form or after coating with DOPE and DOTAP dissolved in mineral oil, referred to as MitoCoat. (B) Schematic timeline of the experimental procedure. (C–E) Intracellular ATP levels were measured using a luminescence-based assay across 10 experimental groups, categorized by H_2_O_2_ exposure (±H_2_O_2_) and treatment type: (i) control (no treatment), (ii) native (uncoated) mitochondria (5 or 10 μg), and (iii) MitoCoat (DOPE/DOTAP-coated mitochondria, 5 or 10 μg). Across all three cell types—adipose-derived stem cells (ASCs, n = 9), bone marrow mesenchymal stem cells (BM-MSCs, n = 9), and astrocytes (n = 4)—H_2_O_2_ exposure significantly reduced ATP levels compared to PBS controls. In ASCs and astrocytes, MitoCoat treatment, especially at 10 μg under oxidative stress, significantly restored ATP levels reduced by H_2_O_2_ (ASCs: p < 0.0001; astrocytes: p < 0.05). In BM-MSCs, both native and MitoCoat mitochondria tended to improve ATP levels after oxidative stress, but no clear dose-dependent effect was observed, and the 10 μg MitoCoat group did not show the highest ATP recovery. Data are presented as mean ± SD. ns, not significant.

We have confirmed that our modification with DOPE and DOTAP lipids showed the efficiency of incorporation into recipient cells in vitro (Figure 2). This section outlines an application for assessing mitochondrial function in your experiments. Here we show one of the applications how mitochondria modified with DOPE and DOTAP are effective against oxidative stress (Figure 3). Specifically, we present in vitro studies conducted on three different cell types: adipose cells, bone marrow–derived cells, and astrocytes.

20. Plate cells on the clear-bottom black plate (96 well) in 100 μL of culture media.
21. Treat cells with 100 μM of H_2_O_2_ at 37°C for 30 min.
  a. After H_2_O_2_ treatment (30 min), rinse with PBS once, and add culture medium.
  b. Incubate at 37°C for 24 h.
22. Add MitoCoat or non-coated mitochondria (5 or 10 μg/ well) in 10 μL of PBS (total volume of media is 110 μL).
  a. Incubate at 37°C for 24 h.
23. Add 100 μL of CellTiter mixture to each well, and incubate for 10 min.
  a. Before adding CellTiter mixture, wash the cells once with PBS.
  b. Prepare the mixture fresh just before use.
24. Place the plate in the plate reader (SpectraMax M5), set reading area of 96-well plate, and record luminescence (default setting).

***Note:*** Cell numbers for plating is depending on which cells you use. Make sure which kinds of cell you use.

***Note:*** CellTiter mixture are prepared by mixing 50 μL of culture medium and 50 μL of CellTiter-Glo reagent.

***Note:*** You can use any kinds of plate readers which can be detected luminescence.

## Expected outcomes

**Figure 4:**
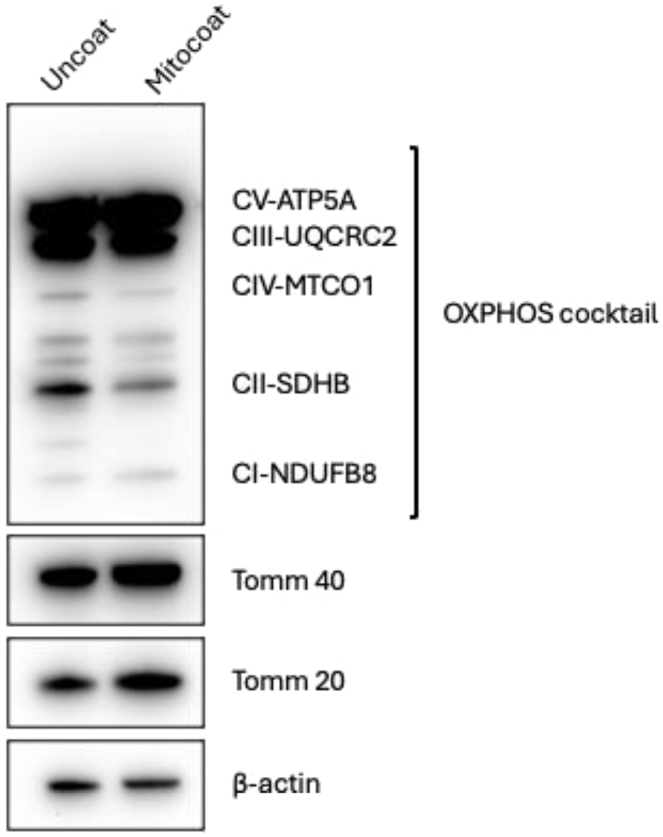
Western blot analysis confirms that lipid coating does not alter the expression of key mitochondrial functional proteins in astrocyte-derived mitochondria To assess whether lipid nanoparticle coating affects mitochondrial integrity, the expression levels of representative mitochondrial proteins were compared between native (uncoated) mitochondria and lipid-coated mitochondria (MitoCoat). No significant differences were observed, indicating that the MitoCoat procedure preserves the integrity of mitochondrial functional components.

Our protocol enables mitochondrial researchers to efficiently modify mitochondria using the MitoCoat strategy. There are no significant differences between native and lipid-coated mitochondria in terms of the protein levels of key mitochondrial functional molecules (Figure 4). In addition to Figure 2 and results of western blot, lipid-coated mitochondria demonstrated the efficiency of mitochondria incorporation into cells and a superior ability to restore ATP levels in damaged cells compared to native mitochondria. These findings indicate that the dual-tube method facilitates straightforward mitochondrial modification while enhancing the restoration of cellular energy production. Furthermore, we have shown that MitoCoat amplified the therapeutic efficiency using stroke model mice in vivo (Nakano et al.^1^). Taken together, this dual system for mitochondrial modification offers a convenient and user-friendly approach for mitochondrial researchers, and can be readily applied to both in vivo and in vitro experiments focused on mitochondrial transfer or transplantation.

## Limitations

Our protocol outlines a method for modifying mitochondria via lipid coating to enhance the therapeutic efficacy of mitochondrial transplantation. However, this approach has not been tested with mitochondrial quantities exceeding 100 μg, and the system requires further optimization for scalability to larger amounts.

**Figure 5:**
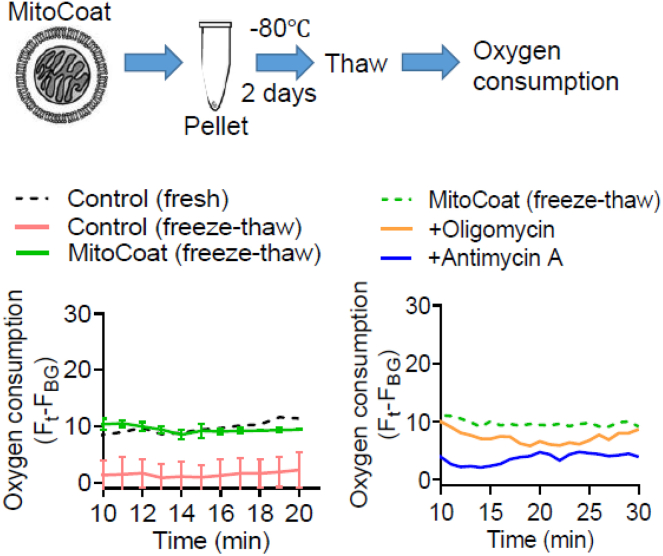
Evaluation of mitochondrial respiratory function after lipid nanoparticle encapsulation and storage Mitochondria were isolated from HEK293 cells, and 50 μg of mitochondrial protein was encapsulated within lipid nanoparticles composed of DOTAP and DOPE. Lipid-coated mitochondria were stored at −80 °C for 2 days before assessing basal oxygen consumption using an oxygen consumption rate (OCR) assay kit (Cayman Chemicals, 600800). Freshly isolated mitochondria, freeze-thawed mitochondria, and freeze-thawed lipid-coated mitochondria were seeded into clear-bottom black 96-well plates. An oxygen sensor probe (10 μL) was added to each well, followed by overlaying with 100 μL of oil. Plates were read at 30°C using excitation and emission filters set at 340 nm and 642 nm, respectively. During OCR measurement, mitochondria were treated with oligomycin (1 μM) or antimycin A (1 μM) to assess the impact of ATP synthase inhibition and electron transport blockade, respectively.

We recommend using freshly isolated mitochondria for lipid coating and preparing the lipid-coated mitochondria immediately prior to use. Although long-term storage has not been extensively evaluated, we examined whether lipid-coated mitochondria (referred to as the MitoCoat group) retained their basal oxygen consumption levels after storage at −80°C for two days (Figure 5). Notably, mitochondrial respiratory activity in the lipid-coated group was reduced upon inhibition of F1Fo ATP synthase with oligomycin or by blocking electron transfer at complex III with antimycin A. This suggests that surface modification with lipids may partially preserve mitochondrial respiratory chain complexes.

Based on these findings, lipid-coated mitochondria can be stored at −80°C for at least two days without substantial loss of respiratory function.

## Troubleshooting

### Problem 1: Leakage of lipid mixture from the inner tube after addition

If lipid mixture is not carefully added to the inner tube (step 7), it may leak out and spread onto the PBS layer in the outer tube, which can compromise the system and be difficult to clean up. This may negatively affect the experimental outcome.

### Potential solution

Ensure that the diameter of inner tube is correct. Also, ensure the lipid nanoparticle layer is properly layered over the PBS in the inner tube (step 6, a). The ratio of PBS and lipid mixture layer is critical.

### Problem 2: Leakage of the lipid mixture or water/oil (W/O) emulsion containing the mitochondria after centrifugation

As mentioned above, if oily substances inside the inner tube—such as the lipid layer or the mitochondria-containing water/oil (W/O) emulsion—leak out after centrifugation, they may compromise the results and are difficult to remove them completely.

### Potential solution

After adding mitochondria mixture onto DOPE/DOTAP layer in the inner tube, ensure that the lid of inner tube is closed completely. Additionally, after centrifugation, carefully lift the inner tube straight up from the outer tube.

### Problem 3: Failure of encapsulation by lipid coating

Although uncoated mitochondria can be functional, lipid-coated mitochondria are more effective in enhancing viability and transfer efficiency.

### Potential solution

The ratio of oil and DOPE and DOTAP is critical for lipid coating processes (detailed in Nakano et al.^1^). Additionally, prepare the suspension buffer fresh before use; otherwise, the proportion of lipid-coated mitochondria will decrease.

### Problem 4: Decrease in protein concentration in the pellet after the MitoCoat process

A decrease in protein concentration in the pellet after centrifugation may occur. This is likely due to the exclusion of fragmented mitochondrial parts during the process. Therefore, the yield of coated mitochondria in the pellet depends on the health and integrity of the isolated mitochondria.

### Potential solution

Ensure the health and integrity of the isolated mitochondria before performing the MitoCoat process. Additionally, as mentioned earlier, use a solution with a higher specific gravity than PBS—such as a mitochondrial suspension buffer containing sucrose—to improve the process.

### Problem 5: Pellet is too hard and difficult to resuspend in PBS

As mentioned above, centrifugation for 10 min at 8,000 × g is sufficient to precipitate coated mitochondria and collect the pellet at the bottom of the tube. If the pellet is too hard to resuspend, consider the following solutions to address the issue.

### Potential solution

If the pellet is too hard to resuspend, consider reducing the centrifugation speed and duration. Additionally, ensure that polyvinyl alcohol (PVA) is properly added to the mitochondrial suspension buffer, as PVA is critical for softening the pellet.

## Resource availability

### Lead contact

Further information and requests for resources and reagents should be directed to and will be fulfilled by the lead contact, Kazuhide Hayakawa, Ph.D. (khayakawa1@mgh.harvard.edu).

### Technical contact

Further technical information should be directed to and will be fulfilled by the technical contact, Gen Hamanaka, Ph.D. (ghamanaka@mgh.harvard.edu) or Dong Bin Back, Ph.D. (dback3@mgh.harvard.edu).

### Materials availability

This study did not generate new unique reagents.

### Data and code availability

• All data reported in this paper will be shared by the lead contact upon request.
• Any additional information required to reanalyze the data reported in this paper is available from the lead contact upon request.

## Acknowledgments

This work was supported in part by grants from the NIH and MGH ECOR Interim Support Fund. AI tools have been used solely for English Editing purposes during the final review of the manuscript.

## Author contributions

G.H., D.B.B., and K.H. conceived the work. G.H. and D.B.B. performed the experiments. G.H. and D.B.B. contributed to visualization and prepared and assembled the figures. G.H., D.B.B., and K.H. wrote the manuscript. K.H. was involved in funding acquisition. G.H., D.B.B., S.I., T.N., and K.H. provided critical reading and scientific discussions. All authors reviewed and approved the final version of this manuscript.

## Declaration of interests

The authors declare no competing interests.
